# Disorders Specifically Associated With Stress in ICD-11

**DOI:** 10.32872/cpe.9711

**Published:** 2022-12-15

**Authors:** Andreas Maercker, David J. Eberle

**Affiliations:** 1 Department of Psychology, Division of Psychopathology and Clinical Intervention, University of Zurich, Zurich, Switzerland; Philipps-University of Marburg, Marburg, Germany

**Keywords:** disorders specifically associated with stress, ICD-11, posttraumatic stress disorder, complex posttraumatic stress disorder, prolonged grief disorder, adjustment disorder

## Abstract

**Background:**

After almost three decades of ICD-10 use for diagnostic purposes, the World Health Organization has conducted a systematic and elaborate evaluation to revise the classification of mental disorders in this system. This revision resulted in the 11th version (ICD-11), introduced in 2022. As one new feature, the ICD-11 forms a new grouping of mental disorders specifically associated with stress.

**Method:**

The current review presents an overview of the diagnostic features and cultural specifications of disorders specifically associated with stress. This grouping includes posttraumatic stress disorder and complex posttraumatic stress disorder, prolonged grief disorder, adjustment disorder, as well as two diagnoses for children, reactive attachment disorder and disinhibited social engagement disorder.

**Results:**

Overall, there is evidence for the improved clinical utility and applicability of these disorders. The disorders have been defined in a parsimonious way by few features, but they suffice for scientific purposes as well.

**Conclusion:**

However, more research is needed to evaluate assessments for the diagnoses and diagnostic features in the ICD-11.

## Highlights

- In the area of trauma and stress, there are two newly specified diagnoses and further redefinitions of the content of the existing diagnoses.
- The ICD-11 features a new grouping of disorders specifically associated with stress.

For almost 30 years, the 10th version of the International Classification of Diseases (ICD-10) was the standard in diagnosing physical diseases as well as mental disorders around the globe. On 1 January 2022, the World Health Organization (WHO) introduced the 11th revision of this diagnostic system and set a new milestone in the classification of mental disorders. Back in 2011, the WHO had appointed several international working groups for revising the section on mental disorders in the ICD-10. One of these working groups was commissioned to create the grouping of *d*iagnoses *s*pecifically *a*ssociated with *s*tress (DSAS). For the development of the 11th revision of the ICD, the ICD-11, the WHO placed particular emphasis on improving the clinical utility and applicability of the diagnoses.

For DSAS, several methodological preparations for the general revision of the ICD-11 were particularly important. For instance, several global mental health surveys were conducted to assess the needs of psychologists and psychiatrists regarding mental health diagnoses (Evans et al., 2013; Reed et al., 2011, 2013). These preliminary mental health surveys concluded that there is a considerable need among health care professionals to create scientifically based diagnoses for stress-related phenomena like complex trauma and pathological grief reactions (Robles et al., 2014). The advisory board of the WHO therefore expected the international working group on DSAS to further evaluate these stress-related phenomena.

Researchers and clinicians with a broad global distribution took part in the working group for DSAS, from Africa (Lynne M. Jones, Ashraf Kagee), America (Marylene Cloitre, Cecile Rousseau), Asia and Australia (Asma Humayan, Daya Somasundaram, Yuriko Suzuki, Richard Bryant), and Europe (Chris Brewin, Andreas Maercker, Simon Wessely), as well as members from global organizations such as the WHO (Michael B. First, Mark van Ommeren, Geoffrey Reed) and the International Committee of the Red Cross (Renato Souza). This composition of experts was chosen to ensure a global applicability of the diagnostic criteria for the new disorders in consideration.

For the proposed mental disorders of the ICD-11 and specifically for DSAS, a comprehensive clinical evaluation was conducted. Between the start of the working group and the final implementation of the ICD-11, several evaluation steps were implemented:

- Diagnostic propositions of the working group for disorders specifically associated with stress were published and discussed in scientific journals (e.g., Maercker et al., 2013) and in the Global Clinical Practice Network^1^1https://gcp.network.
- For the entire ICD-11 section of mental disorders, approximately 20 working groups worked on different disorder groupings as well as cross-sectional features. Each working group developed clinical best practices, organized regional meetings with health care professionals, and consulted local patient representatives for a comprehensive validation of the working groups’ proposals.
- 13 different research centres across the globe implemented clinical case studies to finalize the new disorder and symptom characterizations (Reed et al., 2018).
- More detailed clinical descriptions and diagnostic recommendations (CDDR) for individual disorders were developed. For the CDDR, the WHO pursued an open access approach. Complementary to the frozen release of diagnostic features, the WHO published open access descriptions to implement future diagnostic changes^2^2https://icd.who.int/dev11/l-m/en#/.

As a major aspect of all revisions, the complexity of mental disorder’s characteristics was reduced. For this purpose, previous disorder subtypes were erased or limited (see Reed, 2010). Furthermore, only symptoms with a particular sensitivity and specificity were implemented as diagnostic features. As a consequence, the clinical utility and applicability of ICD-11 diagnoses was significantly improved. Regarding DSAS, the expert group also discussed the inclusion of diagnoses such as embitterment disorder, burnout, continuous trauma disorder, and a more pronounced relation to – or even inclusion of – dissociative disorders. However, these proposals were not realized in the ICD-11. Furthermore, the diagnosis of an acute stress reaction was moved to the ICD-11 section ‘Factors influencing health status’, as such reactions are considered to be normal and are expected to be resolved within a short period after experiencing an aversive life event.

## Disorders Specifically Associated With Stress in Adults

Table 1 presents an overview of disorders specifically associated with stress in the ICD-11 and the corresponding stress-related disorders in the ICD-10 and the DSM-5 (APA, 2013). The diagnostic features of the ICD-11 diagnoses will be outlined in the following sections.

**Table 1:** Disorders Related to Stress and Trauma According to the ICD-11, the ICD-10, and the DSM-5

ICD-11	ICD-10	DSM-5
6B40: Posttraumatic stress disorder	F43.1: Posttraumatic stress disorder	309.81: Posttraumatic stress disorder
6B41: Complex posttraumatic stress disorder	F62.0: Enduring personality change after catastrophic experience	–
6B42: Prolonged grief disorder	–	–
6B43: Adjustment disorder	F43.2X: Adjustment disorders	309.X: Adjustment disorders
6B4Y & 6B4Z: Other specified or unspecified disorders specifically associated with stress	F43.8 & F43.9: Other specified or unspecified reactions to severe stress	309.89 & 309.9: Other specified or unspecified trauma and stressor-related disorders
QE84: Acute stress reaction (in subchapter 24 – no longer a diagnostic entity but a ‘factor influencing health status’)	F43.0: Acute stress reaction	308.3: Acute stress disorder

### Posttraumatic Stress Disorder

For this category, there was essentially a revision and tightening up of the previous definition. Posttraumatic stress disorder (PTSD) may develop after experiencing an extremely distressing or life-threatening event or series of events, such as sexual abuse or a serious accident (WHO, 2022). A core symptom of PTSD is the re-experiencing of the aversive life event in vivid memories. In most cases, such intrusive re-experiencing manifests as flashbacks or nightmares. However, intrusive symptoms can also involve other modalities or body-related re-experiencing, so that odours, sentiments, or other sensations from the traumatic event may be experienced again. Intrusive re-experiencing typically occurs in combination with strong and overwhelming emotions such as fear or horror (see Bar-Haim et al., 2021). In the ICD-11, repetitive or burdensome thinking of the experienced traumatic event is no longer considered to be a manifestation of intrusive re-experiencing as part of a PTSD. Repetitive thoughts have also been found to be characteristic of resilient trauma survivors. Even though remembering the traumatic event might be distressing for these individuals, such thoughts are not specifically associated with PTSD.

The second symptom feature of PTSD is avoidance of memories, activities, situations, or people related to the traumatic event. Importantly, this avoidance behaviour is deliberately produced by the affected individuals. In past conceptualizations, PTSD has sometimes been associated with amnesia as an unconscious avoidance strategy. Such phenomena are no longer part of the avoidance symptoms in the ICD-11, as they rarely occur and are not consciously reflected by affected individuals. In addition, symptoms such as numbing, diminished interest, and emotional alienation have been removed from avoidance definitions, as they are understood as manifestations of comorbid depressive symptoms.

The third symptom group of PTSD consists of persistent perceptions of current heightened threat. Such perceptions may manifest as hypervigilance or enhanced startled reactions to stimuli such as unexpected noises. Due to their unspecific relation to PTSD, hyperarousal phenomena such as disturbed sleep, concentration problems, and increased irritability are no longer listed as PTSD symptoms in the ICD-11.

As for all disorders specifically associated with stress, PTSD is characterized by a significant impairment in personal, social, educational, occupational, or other important areas of functioning. However, some affected individuals are able to maintain a normal level of functioning, which is only possible through considerable psychological and physical effort. Importantly, clinicians need to account for such compensatory behaviours in the diagnostic process to adequately assess the impairment level of an individual (see also Rodriguez et al., 2012).

PTSD typically emerges within several weeks after experiencing the traumatic life event, but it is possible for PTSD symptoms to emerge many months or years after the traumatic life experience. The ICD-11 includes the possibility of delayed onset of PTSD symptoms, without specifying this phenomenon as a subtype. However, no time limit is introduced for this feature because specific time limits do not accurately reflect psychological processes (see Reed et al., 2018). Furthermore, the ICD-11 no longer defines specific stressor characteristics of the traumatic life event, as it has been shown that the type of trauma is not particularly decisive for the subsequent psychopathology. There is empirical evidence showing that the described pattern of PTSD symptoms only occurs in traumatized individuals, thus allowing a reliable differentiation of individuals with and without PTSD (Berntsen et al., 2003; Brewin et al., 2009). It can therefore be strongly assumed that the symptom pattern in the ICD-11 sufficiently describes the phenomenology of PTSD without the inclusion of stressor types.

The ICD-11 features a particular focus on the cultural characteristics of mental disorders. In the case of PTSD, the ICD-11 states that symptoms such as increased anger, headaches, intensified nightmares, or somatic symptoms might occur with different prevalence in certain cultural groups. The ICD-11 also specifies that intrusive re-experiencing is not considered as something unusual in all cultures; rather, it might be seen as an intense but normal way of remembering a critical life event. Furthermore, certain symptoms can also trigger dysfunctional health beliefs. For instance, anxiety-related symptoms such as persistent perceptions of heightened current threat might be interpreted as a lifelong condition of weak nerves or a weak heart, as is sometimes observed in Latin American countries or in Cambodia. All these aspects need to be considered when working with individuals from different cultural groups.

### Complex Posttraumatic Stress Disorder

Complex posttraumatic stress disorder (CPTSD) may develop after experiencing a traumatic life event that is particularly horrific or threatening (WHO, 2022). In most cases, the stressor consists of a series of traumatic situations or an ongoing event, such as slavery or repeated abuse. Many psychosocial stressors with an extremely threatening nature have the potential to cause CPTSD. However, as is the case for PTSD, the diagnosis mainly depends on symptomatic presentation instead of specific event characteristics (Maercker et al., 2022).

Regarding the psychopathological features of CPTSD, all symptom requirements of PTSD need to be met, including intrusive re-experiencing, avoidance, and persistent perceptions of heightened current threat. In addition, CPTSD is characterized by disturbances in self-organization (DSO), which is indicated by several symptom patterns. First, DSO features problems in affect regulation, which might manifest as frequent excitability, anger, rage, or an increased self-harming behaviour. Second, individuals with CPTSD exhibit beliefs about the self as worthless, defeated, or diminished, which is often accompanied by feelings of guilt, shame, or failure related to the stressful life event. The third feature of DSO constitutes interpersonal problems. The inability to trust, a susceptibility to hyperbolic views, and difficulties in partnership interactions are particularly characteristic for this symptom group. Individuals with CPTSD also show an increased tendency for dissociation (see also Hyland et al., 2020), which includes depersonalization experiences, clouding of consciousness, and amnesia. Contrary to the DSO symptoms, however, dissociation is not a diagnostic requirement for CPTSD.

The introduction of CPTSD as a new disorder in the ICD-11 generated significant criticism. For instance, one criticism is that CPTSD only represents a comorbidity between PTSD and borderline personality disorder, which makes an introduction of a new disorder redundant (Resick et al., 2012, see Maercker, 2021). However, empirical findings demonstrated that CPTSD possesses a distinct, reliable, and useful symptom profile (Brewin et al., 2017; Kazlauskas et al., 2018), which finally led to the inclusion of CPTSD in the ICD-11. In the ICD-10, CPTSD was classified as an enduring personality change after catastrophic experiences. However, continuous research showed that the related symptomatic features were part of a posttraumatic syndrome, which is why this psychopathological type has been reallocated to disorders specifically associated with stress.

According to the ICD-11, CPTSD also exhibits an important cultural variation. In particular, dissociative and somatic symptoms are believed to increasingly emerge in certain cultural groups. Furthermore, migrants across the globe are of particular concern in trauma sequelae. As they are frequently and often repeatedly confronted with severely stressful life events, migrants have a highly increased prevalence of suffering from CPTSD. When migrating to countries with a different cultural background, CPTSD might be triggered and intensified by the ongoing stressors experienced related to migration. As refugees are sometimes faced with continuous violence or discrimination in host countries, they represent a group that is particularly vulnerable to severe disorders specifically associated with stress. Even though research has not yet identified a distinct set of cultural properties of CPTSD, recent publications have started to shed light on these characteristics (see Heim et al., 2022).

### Prolonged Grief Disorder

Compared to other disorders specifically associated with stress, stressors leading to a prolonged grief disorder (PGD) are defined more precisely. PGD might develop after the death of a loved person, such as a partner, parent, child, other family member, or another person close to the bereaved (WHO, 2022). Importantly, animals are not included in this definition. The event of loss causes an intense and long-lasting grief reaction, which can take on many individually different manifestations. However, in terms of common symptoms, PGD is defined by intensive yearning and longing for the deceased, as well as by intrusive preoccupation with the death of the loved person or the implications of this event. In addition to these core symptoms, the ICD-11 defines several accessory symptoms, including guilt, sadness, denial, anger, blame, difficulty accepting the loss, an inability to be in a positive mood, numbness, and a diminished interest in activities. However, the ICD-11 does not define the number of accessory symptoms needed for a PGD diagnosis.

More cultural characteristics are specified for PGD than for other mental disorders. Cultural practices and attitudes towards bereavement strongly differ across the globe. Ideas and concepts of the afterlife manifest a broad range of clinical presentations and behaviours related to bereavement, which may also increase the chance for a prolongation of grief. For instance, the ICD-11 states that in some religions, death is regarded as an important step in the transition to the afterlife. Cultural beliefs focusing on rebirth, but also on karma, heaven, or hell, can have an enormous impact on a bereaved person. PGD might therefore be additionally triggered by concerns about the afterlife of the deceased. According to some religious beliefs, such as those common in southern Europe, an encounter with the spirit of a deceased person – which may be regarded as a symptom of re-experience – is not considered as an abnormal event and may even be perceived as a positive experience. Another culturally diverse feature in relation to PGD is the duration of grief, as there are different norms across the globe concerning mourning periods. In some countries, a one-year mourning period is considered as normal, whereas in other cultures, mourning periods are considered to trigger negative emotions and are therefore kept relatively short.

Due to these various cultural manifestations, the ICD-11 states that for the diagnosis of PGD, the cultural background of patients needs to be evaluated thoroughly. The diagnosis of PGD should only be made if the grief reaction clearly exceeds the respective cultural norms of the individual. In general, the ICD-11 states that PGD may be diagnosed no earlier than six months after the death of the loved person. However, due to the cultural variations outlined before, the duration of grief should correspond to the cultural background when considering a PGD diagnosis. Long-lasting grief reactions that are still within a cultural norm are classified as a normal grief reaction and not as PGD. The extent to which different cultures affect the expression of symptoms remains the subject of further research.

There were also some objections to the introduction of PGD as a new IDC-11 diagnosis. For instance, one criticism was that the introduction of PGD as a new diagnosis represents disease mongering and that grief should always be classified as a natural process of life. However, it should be noted that in the past, prolonged grief has mostly been falsely diagnosed as depression, PTSD, or adjustment disorder, even for the small number of those it affects. Such diagnoses are not only clinically inaccurate but can also cause inadequate treatment. For individuals affected by mental disorders, a diagnosis can be helpful to understand and address psychological problems, presupposing that the underlying problems are correctly identified in the first place.

### Adjustment Disorder

Another disorder specifically associated with stress is adjustment disorder (AjD). This disorder may develop after one or several critical life event(s), such as involuntary job loss, severe illness, or a relationship breakup (WHO, 2022). On a symptomatic level, AjD is characterized by an intrusive preoccupation with the aversive life event or its implications, which mainly manifests as repetitive and distressing thoughts of the event. Failure to adapt constitutes a further AjD symptom, which may take the form of sleep and concentration problems or an inability to recuperate. Due to the high levels of distress that individuals with AjD experience, suicidal tendencies are not uncommon as part of the disorder. Importantly, the diagnosis of AjD specifies that disorder-related symptoms persist no longer than six months after the aversive life event. However, in the case of a prolonged exposure to a stressor, such as an ongoing illness, AjD may also be diagnosed for longer than six months.

In general, all aversive life events have the potential to trigger AjD, which makes it particularly difficult to differentiate such experiences from traumatic events and sequelae. However, a great majority of individuals diagnosed with PTSD and CPTSD have been confronted with life-threatening experiences, whereas events leading to AjD are not particularly overwhelming in most cases. Even though stressors like a divorce might be extremely stressful for those affected, such events are usually not associated with a threat to one’s core identity and basic tenets of life during exposure to the stressor and therefore do not cause typical posttraumatic symptoms (Brewin, 2014; Eberle & Maercker, 2022).

The manifestation of AjD varies across the lifespan. According to the ICD-11, children with AjD may typically exhibit increased disruptive or oppositional behaviour, hyperactivity, irritability, concentration problems, increased clinginess, tantrums, regression, sleep disturbances, or bedwetting. In contrast to children, adolescents may manifest an intensification of substance use as well as increased behaviours of acting out or risk taking. Children and adolescents with AjD often fail to verbalize their emotions related to the stressful experience. Therefore, it is important to account for this interactive inhibition in the diagnostic process and relate reports of critical life events to changed behaviour patterns. Meanwhile, older adults diagnosed with AjD increasingly manifest psychosomatic symptoms as a reaction to critical life events. Consequently, in this age group, the core AjD symptom of preoccupation is especially focused on their own health (for more age-specific information, see also Mulligan, 2018; WHO, 2022).

The ICD-11 states that in some cultural groups, AjD might intensify significantly in the case of lacking family or community support. Furthermore, local idioms of distress and concepts of suffering can play a significant role in the manifestation of AjD. For example, exposure to aversive life events may result in particularly strong anxiety reactions, as it has been observed in individuals from Central America.

## Additional Disorders for Children

In the ICD-11, diagnoses for children and adolescents are no longer separately coded but are rather implemented in the disorder group of the appropriate life-span diagnoses. This means that the grouping of disorders specifically associated with stress also features two diagnoses for children and adolescents: disinhibited social engagement disorder and reactive attachment disorder (WHO, 2022). One childhood-specific stress-related diagnosis listed in the ICD-10 has not been transferred to the ICD-11. Due to the phenomenological overlap, autism spectrum disorder is an important exclusion criterion for both childhood disorders specifically associated with stress in the ICD-11.

Disinhibited social engagement disorder develops as a consequence of grossly inadequate childcare, such as institutional deprivation, severe neglect of the child’s physical or emotional needs, a constant change of primary caregivers, parenting in inadequate settings, and child abuse (see also Zeanah et al., 2016). According to the ICD-11, children with disinhibited social engagement disorder are characterized by an indiscriminate approaching of adults, a lack of restraint to approaching, an overly familiar behaviour towards strangers, and a willingness to go away with unfamiliar adults. Disinhibited social engagement disorder is relatively rare and has been found to develop only in a small proportion of children who have experienced inadequate care.

Reactive attachment disorder, as the second child-specific stress-related disorder in the ICD-11, is also characterized by highly inadequate childcare. The disorder features an inhibited attachment behaviour of the child. According to the ICD-11, this may manifest as an unwillingness to return to the primary caregiver for nurture, comfort, or support, even though an adequate caregiver is available. Furthermore, the child does not respond when comfort is offered and rarely displays security-seeking behaviours towards any adult (Zeanah et al., 2016).

## Questionnaires and Clinical Interviews

With its revised diagnostic features for mental disorders, the ICD-11 also requires an adaptation in the assessment of these disorders. In recent years, new measurement instruments for DSAS have been developed. For the development of these diagnostic assessment tools, a European-American consortium has been founded: the International Trauma Consortium^3^3www.traumameasuresglobal.com, which offers freely available diagnostic instruments in numerous languages. While English versions of the developed scales are already fully validated, the validation processes for other languages, such as German or Arabic, are not yet completed.

## The ICD-11 in Clinical Practice

The new ICD-11 diagnoses have been repeatedly evaluated. For instance, various disorders have been cross-compared with mental health conceptualizations from the ICD-10 and the DSM-5, as will be shown in the following paragraphs. However, with regard to prevalence studies, data sets based on epidemiological and high-risk samples often cover individuals who are not in treatment. Therefore, studies with patients undergoing actual treatment are most relevant for an evaluation of the ICD-11 in clinical practice. In addition, many previous studies have not assessed the diagnostic features of impairment in personal, family, social, educational, occupational, or other important areas of functioning, even though this feature is a critical diagnostic element. These limitations need to be kept in mind when diagnostic findings are compared. Regarding childhood disorders, studies have not yet managed to replicate the prevalence numbers of the disorders, which is why the following section will not evaluate disinhibited social engagement disorder and reactive attachment disorder.

### PTSD and CPTSD

Regarding PTSD, the first study to evaluate different diagnostic systems involving the ICD-11 was conducted as part of the world mental health surveys (Stein et al., 2014). The assessment applying the ICD-11 indicated that 3.2% of screened individuals met the diagnostic criteria for PTSD. In comparison, a prevalence of 4.4% was found with the ICD-10 and a prevalence of 3.0% was found with the DSM-5. Among all individuals who received a PTSD diagnosis with the ICD-11, the ICD-10, or the DSM-5, 75% were diagnosed accordingly in all three classification systems. Another study including a high-risk sample of older adults found a PTSD prevalence of 10.3% when diagnosed with the ICD-11. In comparison, according to the ICD-10, 15% of individuals met all diagnostic features of PTSD (Glück et al., 2016).

Prevalence numbers differ for more specific populations, such as members of the military. Wisco et al. (2016) found that, in a high-risk sample of US military personnel, 34% were diagnosed with PTSD according to the ICD-11, while 45% were diagnosed with the ICD-10 and 34% with the DSM-5. The diagnostic overlap between the ICD-11 and the DSM-5 was 89%. A similar study has been conducted in the German military: Kuester et al. (2017) found PTSD rates of 48% for the ICD-11, 30% for the ICD-10, and 56% for the DSM-5. The diagnostic overlap between the ICD-11 and the DSM-5 was 84%. However, both of these studies only used validated DSM instruments for their assessment, which were adapted to also capture ICD diagnoses. Furthermore, Møller et al. (2020) investigated PTSD and CPTSD in a patient sample. Of the patients who received a PTSD diagnosis according to the ICD-10, 46% were also diagnosed with PTSD according to the ICD-11, 28% were diagnosed with CPTSD, and 26% were diagnosed with another mental disorder.

In summary, empirical studies show that the diagnostic overlap between different classification systems must be estimated at roughly 60–90%. In clinical practice, this means that even though a patient might receive a PTSD diagnosis according to the ICD-10 or the DSM-5, a PTSD diagnosis may no longer be assigned when using the ICD-11. Such empirical findings might seem upsetting, as all diagnostic systems are supposed to ensure valid diagnostic results. However, it must be considered that diagnostic tools are always subject to a minimal level of uncertainty, which may lead to different results. Furthermore, the theoretical background for diagnostic characteristics have changed between different classification systems. For instance, symptoms of re-experience have been laid out more strictly in the ICD-11. If an individual exhibits distressing repetitive thoughts of a trauma but no vivid flashbacks or severe nightmares, the diagnosis of PTSD is no longer indicated by the ICD-11.

### AjD and PGD

Prevalence numbers for both AjD and PGD are not yet conclusively determined due to sparse research activity and changing disorder definitions over the last years. A diagnostic evaluation based on the ICD-10 found that across different countries, AjD exhibits a prevalence of approximately 1% (Ayuso-Mateos et al., 2001). This finding was replicated in a German study by Maercker et al. (2012), which found an AjD prevalence of 0.9% by implementing ICD-11 features. Therefore, in contrast to other disorders specifically associated with stress, AjD appears to show little variability in the prevalence figures of the different diagnostic systems. Since PGD was newly introduced in the ICD-11, no comparison with broadly established conceptualizations of grief is possible. However, the DSM-5 defines persistent complex grief disorder as a research diagnosis. Maciejewski et al. (2016) compared this diagnosis with the ICD-11 definition and found a kappa coefficient of 0.82, which indicates a big overlap between the two disorders. Importantly, in the upcoming DSM-5-TR, PGD will be included as a regular disorder in the classification system (Moran, 2021). Hence, it is hoped that future research will be able to conduct thorough comparisons between classification systems and adequate prevalence estimations.

## Conclusion

Disorders specifically associated with stress encompass a set of psychopathological sequelae emerging after exposure to a stressful life event. Research shows that these revised disorders entail an increased clinical utility and applicability. However, more studies are needed to investigate the long-term benefits of the new DSAS grouping of disorders. It is hoped that the ICD-11, which will guide clinicians and their therapeutic actions over the next decades, proves to be beneficial for individuals suffering mental disorders from the kinds of external sources outlined here. We may see further steps towards convergence with the DSM-5 as well, such as with PGD, which was included in the DSM-5-TR (**t**ext **r**evision) in 2022.
